# Supplementary material for: Evolutionary divergence reveals the molecular basis of EMRE dependence of the human MCU
Source: Life Sci Alliance. 2020 Aug 7;3(10):e202000718. doi: 10.26508/lsa.202000718 (PMC7425227; doi:10.26508/lsa.202000718)
Supplement: Supplementary file 1 [file LSA-2020-00718_Supplemental_Data_1.docx]

**SUPPLEMENTARY MATERIALS**

*H. sapiens* DNA and amino acid sequences are in UPPERCASE characters.

*D. discoideum* or *C. elegans* DNA and amino acid sequences are **in lowercase, bold characters**.

For MCU chimeras, chimera breakpoints are highlighted in red.

For each construct, both DNA sequence and amino acid sequence are shown

FLAG tag sequence is highlighted in green.

3X GGS linker sequence between MCU and FLAG tag is highlighted in blue.

**HsMCU:**

ATGGCGGCCGCCGCAGGTAGATCGCTCCTGCTGCTCCTCTCCTCTCGGGGCGGCGGCGGCGGGGGCGCCGGCGGCTGCGGGGCGCTGACTGCCGGCTGCTTCCCTGGGCTGGGCGTCAGCCGCCACCGGCAGCAGCAGCACCACCGGACGGTACACCAGAGGATCGCTTCCTGGCAGAATTTGGGAGCTGTTTATTGCAGCACTGTTGTGCCCTCTGATGATGTTACAGTGGTTTATCAAAATGGGTTACCTGTGATATCTGTGAGGCTACCATCCCGGCGTGAACGCTGTCAGTTCACACTCAAGCCTATCTCTGACTCTGTTGGTGTATTTTTACGACAACTGCAAGAAGAGGATCGGGGAATTGACAGAGTTGCTATCTATTCACCAGATGGTGTTCGCGTTGCTGCTTCAACAGGAATAGACCTCCTCCTCCTTGATGACTTTAAGCTGGTCATTAATGACTTAACATACCACGTACGACCACCAAAAAGAGACCTCTTAAGTCATGAAAATGCAGCAACGCTGAATGATGTAAAGACATTGGTCCAGCAACTATACACCACACTGTGCATTGAGCAGCACCAGTTAAACAAGGAAAGGGAGCTTATTGAAAGACTAGAGGATCTCAAAGAGCAGCTGGCTCCCCTGGAAAAGGTACGAATTGAGATTAGCAGAAAAGCTGAGAAGAGGACCACTTTGGTGCTATGGGGTGGCCTTGCCTACATGGCCACACAGTTTGGCATTTTGGCCCGGCTTACCTGGTGGGAATATTCCTGGGACATCATGGAGCCAGTAACATACTTCATCACTTATGGAAGTGCCATGGCAATGTATGCATATTTTGTAATGACACGCCAGGAATATGTTTATCCAGAAGCCAGAGACAGACAATACTTACTATTTTTCCATAAAGGAGCCAAAAAGTCACGTTTTGACCTAGAGAAATACAATCAACTCAAGGATGCAATTGCTCAGGCAGAAATGGACCTTAAGAGACTGAGAGACCCATTACAAGTACATCTGCCTCTCCGACAAATTGGTGAAAAAGATTCTAGAGGTGGATCTGGTGGATCTGGTGGATCTATGGATTACAAGGATGACGATGACAAG*

MAAAAGRSLLLLLSSRGGGGGGAGGCGALTAGCFPGLGVSRHRQQQHHRTVHQRIASWQNLGAVYCSTVVPSDDVTVVYQNGLPVISVRLPSRRERCQFTLKPISDSVGVFLRQLQEEDRGIDRVAIYSPDGVRVAASTGIDLLLLDDFKLVINDLTYHVRPPKRDLLSHENAATLNDVKTLVQQLYTTLCIEQHQLNKERELIERLEDLKEQLAPLEKVRIEISRKAEKRTTLVLWGGLAYMATQFGILARLTWWEYSWDIMEPVTYFITYGSAMAMYAYFVMTRQEYVYPEARDRQYLLFFHKGAKKSRFDLEKYNQLKDAIAQAEMDLKRLRDPLQVHLPLRQIGEKDSRGGSGGSGGSMDYKDDDDK*

**DdMCU:**

**atgaatagcttcgtgatcaggaacggcttcggcctcgtcaggaccttcaacaccaggctgtttaccacctccacccagaacctggagggcgagctcaaaaccatcctcggccaggccaaggtcagcaagctgcaggaaaagctgaagctggatcccaggtccaagatcaccttcaacgacttcaagggcatcgccaaggaggtgggcatcgaggagaaggaaatcaacagcgtcagcaacgccctggctcaatccggctccatcatctacctgcctaactccctgaacgagaacctgaagacctccgtgttcaccaagcccgcccatatctaccagtccctcgaacacatcctggacatcgagaacaagggcgtgggcctgaataagctgatcgagtccaagaaatccgagatcaatagcctgaggcagaagatccagcccctggaggagaagaagcaggtgatcgacagaaaggcccacaggagggccaccgctatcatctggaccggactcggctattgcttcgcccaggccgccattctggctaggctcacctggtgggacctctcctgggacatcatcgagcccgtgagctacttcctgaccttcggctccgtcctgatcggctacacctacttcaccatgaccaagaccgagttcacctacgaggctctcaaccataggctgttcagcaagaggcaggacaagctgttcaaaagaaacaacttccctaaggaggactacgagaacctcgtgcaggccatcgacaagaaggagaaggagctcaaggagctcgagctcgccacaaagtacgatcacacccaccccggg**GGTGGATCTGGTGGATCTGGTGGATCTATGGATTACAAGGATGACGATGACAAG*****

**mnsfvirngfglvrtfntrlfttstqnlegelktilgqakvsklqeklkldprskitfndfkgiakevgieekeinsvsnalaqsgsiiylpnslnenlktsvftkpahiyqslehildienkgvglnklieskkseinslrqkiqPleekkqvidrkahrRataiiwtglgycfaqaailarltwwdlswdiiepvsyfltfgsvligytyftmtktEftyealnhRlfskrqdKlfkrnnFpkedyenlvqaidkkekelkelelatkydhthpg**GGSGGSGGSMDYKDDDDK*

**Chimera 1**

**atgaatagcttcgtgatcaggaacggcttcggcctcgtcaggaccttcaacaccaggctgtttaccacctccacccagaacctggagggcgagctcaaaaccatcctcggccaggccaaggtcagcaagctgcaggaaaagctgaagctggatcccaggtccaagatcaccttcaacgacttcaagggcatcgccaaggaggtgggcatcgaggagaaggaaatcaacagcgtcagcaacgccctggctcaatccggctccatcatctacctgcctaactccctgaacgagaacctgaagacctccgtgttcaccaagcccgcccatatctaccagtccctcgaacacatcctggacatcgagaacaagggcgtgggcctgaataagctgatcgagtccaagaaatccgagatcaatagcctgaggcagaagatccag**CCCCTGGAAAAGGTACGAATTGAGATTAGCAGAAAAGCTGAGAAGAGGACCACTTTGGTGCTATGGGGTGGCCTTGCCTACATGGCCACACAGTTTGGCATTTTGGCCCGGCTTACCTGGTGGGAATATTCCTGGGACATCATGGAGCCAGTAACATACTTCATCACTTATGGAAGTGCCATGGCAATGTATGCATATTTTGTAATGACACGCCAGGAATATGTTTATCCAGAAGCCAGAGACAGACAATACTTACTATTTTTCCATAAAGGAGCCAAAAAGTCACGTTTTGACCTAGAGAAATACAATCAACTCAAGGATGCAATTGCTCAGGCAGAAATGGACCTTAAGAGACTGAGAGACCCATTACAAGTACATCTGCCTCTCCGACAAATTGGTGAAAAAGATCCCGGGGGTGGATCTGGTGGATCTGGTGGATCTATGGATTACAAGGATGACGATGACAAG*

**mnsfvirngfglvrtfntrlfttstqnlegelktilgqakvsklqeklkldprskitfndfkgiakevgieekeinsvsnalaqsgsiiylpnslnenlktsvftkpahiyqslehildienkgvglnklieskkseinslrqkiq**PLEKVRIEISRKAEKRTTLVLWGGLAYMATQFGILARLTWWEYSWDIMEPVTYFITYGSAMAMYAYFVMTRQEYVYPEARDRQYLLFFHKGAKKSRFDLEKYNQLKDAIAQAEMDLKRLRDPLQVHLPLRQIGEKDPGGGSGGSGGSMDYKDDDDK*

**Chimera 2**

**atgaatagcttcgtgatcaggaacggcttcggcctcgtcaggaccttcaacaccaggctgtttaccacctccacccagaacctggagggcgagctcaaaaccatcctcggccaggccaaggtcagcaagctgcaggaaaagctgaagctggatcccaggtacaagatcaccttcaacgacttcaagggcatcgccaaggaggtgggcatcgaggagaaggaaatcaacagcgtcagcaacgccctggctcaatccggctccatcatctacctgcctaactccctgaacgagaacctgaagacctccgtgttcaccaagcccgcccatatctaccagtccctcgaacacatcctggacatcgagaacaagggcgtgggcctgaataagctgatcgagtccaagaaatccgagatcaatagcctgaggcagaagatccagcccctggaggagaagaagcaggtgatcgacagaaaggcccacaggagggccaccgctatcatctggaccggactcggctattgcttcgcccaggccgccattctggctaggctcacctggtgggacctctcctgggacatcatcgagcccgtgagctacttcctgaccttcggctccgtcctgatcggctacacctacttcacnatgaccaagacc**GAATATGTTTATCCAGAAGCCAGAGACAGACAATACTTACTATTTTTCCATAAAGGAGCCAAAAAGTCACGTTTTGACCTAGAGAAATACAATCAACTCAAGGATGCAATTGCTCAGGCAGAAATGGACCTTAAGAGACTGAGAGACCCATTACAAGTACATCTGCCTCTCCGACAAATTGGTGAAAAAGATCCCGGGGGTGGATCTGGTGGATCTGGTGGATCTATGGATTACAAGGATGACGATGACAAG*

**mnsfvirngfglvrtfntrlfttstqnlegelktilgqakvsklqeklkldprykitfndfkgiakevgieekeinsvsnalaqsgsiiylpnslnenlktsvftkpahiyqslehildienkgvglnklieskkseinslrqkiqPleekkqvidrkahrRataiiwtglgycfaqaailarltwwdlswdiiepvsyfltfgsvligytyftmtkt**EYVYPEARDRQYLLFFHKGAKKSRFDLEKYNQLKDAIAQAEMDLKRLRDPLQVHLPLRQIGEKDPGGGSGGSGGSMDYKDDDDK*

**Chimera 3**

ATGGCAGCTGCAGAAGGTAGATCGCTACTGCTACTCCTATCCTCTCGGGGAGGTGGAGGTGGAGGTGCCGGAGGTTGCGGGACGCTGACTGCCGGCTGCTTCCCTGGGCTGGGCGTCAGCCGCCACCGGCAGCAGCAGCACCACCGGACGGTACACCAGAGGATCGCTTCCTGGCAGAATTTGGGAGCTGTTTATTGCAGCACTGTTGTGCCCTCTGATGATGTTACAGTGGTTTATCAAAATGGGTTACCTGTGATATCTGTGAGGCTACCATCCCGGCGTGAACGCTGTCAGTTCACACTCAAGCCTATCTCTGACTCTGTTGGTGTATTTTTACGACAACTGCAAGAAGAGGATCGGGGAATTGACAGAGTTGCTATCTATTCACCAGATGGTGTTCGCGTTGCTGCTTCAACAGGAATAGACCTCCTCCTCCTTGATGACTTTAAGCTGGTCATTAATGACTTAACATACCACGTACGACCACCAAAAAGAGACCTCTTAAGTCATGAAAATGCAGCAACGCTGAATGATGTAAAGACATTGGTCCAGCAACTATACACCACACTGTGCATTGAGCAGCACCAGTTAAACAAGGAAAGGGAGCTTATTGAAAGACTAGAGGATCTCAAAGAGCAGCTGGCTCCCCTGGAAAAGGTACGAATTGAGATTAGCAGAAAAGCTGAGAAGAGG**gccaccgctatcatctggaccggactcggctattgcttcgcccaggccgccattctggctaggctcacctggtgggacctctcctgggacatcatcgagcccgtgagctacttcctgaccttcggctccgtcctgatcggctacacctacttcaccatgaccaagaccgag**TATGTTTATCCAGAAGCCAGAGACAGACAATACTTACTATTTTTCCATAAAGGAGCCAAAAAGTCACGTTTTGACCTAGAGAAATACAATCAACTCAAGGATGCAATTGCTCAGGCAGAAATGGACCTTAAGAGACTGAGAGACCCATTACAAGTACATCTGCCTCTCCGACAAATTGGTGAAAAAGATCCCGGGGGTGGATCTGGTGGATCTGGTGGATCTATGGATTACAAGGATGACGATGACAAG*

MAAAEGRSLLLLLSSRGGGGGGAGGCGTLTAGCFPGLGVSRHRQQQHHRTVHQRIASWQNLGAVYCSTVVPSDDVTVVYQNGLPVISVRLPSRRERCQFTLKPISDSVGVFLRQLQEEDRGIDRVAIYSPDGVRVAASTGIDLLLLDDFKLVINDLTYHVRPPKRDLLSHENAATLNDVKTLVQQLYTTLCIEQHQLNKERELIERLEDLKEQLAPLEKVRIEISRKAEKR**ataiiwtglgycfaqaailarltwwdlswdiiepvsyfltfgsvligytyftmtkt**EYVYPEARDRQYLLFFHKGAKKSRFDLEKYNQLKDAIAQAEMDLKRLRDPLQVHLPLRQIGEKDPGGGSGGSGGSMDYKDDDDK*

**Chimera 4**

**atgaatagcttcgtgatcaggaacggcttcggcctcgtcaggaccttcaacaccaggctgtttaccacctccacccagaacctggagggcgagctcaaaaccatcctcggccaggccaaggtcagcaagctgcaggaaaagctgaagctggatcccaggtccaagatcaccttcaacgacttcaagggcatcgccaaggaggtgggcatcgaggagaaggaaatcaacagcgtcagcaacgccctggctcaatccggctccatcatctacctgcctaactccctgaacgagaacctgaagacctccgtgttcaccaagcccgcccatatctaccagtccctcgaacacatcctggacatcgagaacaagggcgtgggcctgaataagctgatcgagtccaagaaatccgagatcaatagcctgaggcagaagatccagcccctggaggagaagaagcaggtgatcgacagaaaggcccacag**GAGGACCACTTTGGTGCTATGGGGTGGCCTTGCCTACATGGCCACACAGTTTGGCATTTTGGCCCGGCTTACCTGGTGGGAATATTCCTGGGACATCATGGAGCCAGTAACATACTTCATCACTTATGGAAGTGCCATGGCAATGTATGCATATTTTGTAATGACACGCCAGGAAT**tcacctacgaggctctcaaccataggctgttcagcaagaggcaggacaagctgttcaaaagaaacaacttccctaaggaggactacgagaacctcgtgcaggccatcgacaagaaggagaaggagctcaaggagctcgagctcgccacaaagtacgatcacacccaccccggg**GGTGGATCTGGTGGATCTGGTGGATCTATGGATTACAAGGATGACGATGACAAG*

**Combined:**

**mnsfvirngfglvrtfntrlfttstqnlegelktilgqakvsklqeklkldprskitfndfkgiakevgieekeinsvsnalaqsgsiiylpnslnenlktsvftkpahiyqslehildienkgvglnklieskkseinslrqkiqPleekkqvidrkahrR**TTLVLWGGLAYMATQFGILARLTWWEYSWDIMEPVTYFITYGSAMAMYAYFVMTRQ**EftyealnhRlfskrqdKlfkrnnFpkedyenlvqaidkkekelkelelatkydhthpg**GGSGGSGGSMDYKDDDDK*

**Chimera 5:**

ATGGCAGCTGCAGCAGGTAGATCGCTACTGCTACTCCTATCCTCTCGGGGAGGTGGAGGTGGAGGTGCCGGAGGTTGCGGGACGCTGACTGCCGGCTGCTTCCCTGGGCTGGGCGTCAGCCGCCACCGGCAGCAGCAGCACCACCGGACGGTACACCAGAGGATCGCTTCCTGGCAGAATTTGGGAGCTGTTTATTGCAGCACTGTTGTGCCCTCTGATGATGTTACAGTGGTTTATCAAAATGGGTTACCTGTGATATCTGTGAGGCTACCATCCCGGCGTGAACGCTGTCAGTTCACACTCAAGCCTATCTCTGACTCTGTTGGTGTATTTTTACGACAACTGCAAGAAGAGGATCGGGGAATTGACAGAGTTGCTATCTATTCACCAGATGGTGTTCGCGTTGCTGCTTCAACAGGAATAGACCTCCTCCTCCTTGATGACTTTAAGCTGGTCATTAATGACTTAACATACCACGTACGACCACCAAAAAGAGACCTCTTAAGTCATGAAAATGCAGCAACGCTGAATGATGTAAAGACATTGGTCCAGCAACTATACACCACACTGTGCATTGAGCAGCACCAGTTAAACAAGGAAAGGGAGCTTATTGAAAGACTAGAGGATCTCAAAGAGCAGCTGGCTCCCCTGGA**ggagaagaagcaggtgatcgacagaaaggcccacaggagggccaccgctatcatctggaccggactcggctattgcttcgcccaggccgccattctggctaggctcacctggtgggacctctcctgggacatcatcgagcccgtgagctacttcctgaccttcggctcngtcctgatcggctacacctacttcaccatgaccaagaccgagttcacctacgaggctctcaaccataggctgttcagcaagaggcaggacaagctgttcaaaagaaacaacttccctaaggaggactacgagaacctcgtgcaggccatcgacaagaaggagaaggagctcaaggagctcgagctcgccacaaagtacgatcacacccaccccgg**gGGTGGATCTGGTGGATCTGGTGGATCTATGGATTACAAGGATGACGATGACAAG*

MAAAAGRSLLLLLSSRGGGGGGAGGCGTLTAGCFPGLGVSRHRQQQHHRTVHQRIASWQNLGAVYCSTVVPSDDVTVVYQNGLPVISVRLPSRRERCQFTLKPISDSVGVFLRQLQEEDRGIDRVAIYSPDGVRVAASTGIDLLLLDDFKLVINDLTYHVRPPKRDLLSHENAATLNDVKTLVQQLYTTLCIEQHQLNKERELIERLEDLKEQLAPLE**ekkqvidrkahrRataiiwtglgycfaqaailarltwwdlswdiiepvsyfltfgsvligytyftmtktEftyealnhRlfskrqdKlfkrnnFpkedyenlvqaidkkek**elkelelatkydhthpgGGSGGSGGSMDYKDDDDK*

**Chimera 6:**

ATGGCAGCTGCAGCAGGTCGATCGCTACTGCTACTCCTATCCTCTCGGGGAGGTGGAGGTGGAGGTGCCGGAGGTTGCGGGACGCTGACTGCCGGCTGCTTCCCTGGGCTGGGCGTCAGCCGCCACCGGCAGCAGCAGCACCACCGGACGGTACACCAGAGGATCGCTTCCTGGCAGAATTTGGGAGCTGTTTATTGCAGCACTGTTGTGCCCTCTGATGATGTTACAGTGGTTTATCAAAATGGGTTACCTGTGATATCTGTGAGGCTACCATCCCGGCGTGAACGCTGTCAGTTCACACTCAAGCCTATCTCTGACTCTGTTGGTGTATTTTTACGACAACTGCAAGAAGAGGATCGGGGAATTGACAGAGTTGCTATCTATTCACCAGATGGTGTTCGCGTTGCTGCTTCAACAGGAATAGACCTCCTCCTCCTTGATGACTTTAAGCTGGTCATTAATGACTTAACATACCACGTACGACCACCAAAAAGAGACCTCTTAAGTCATGAAAATGCAGCAACGCTGAATGATGTAAAGACATTGGTCCAGCAACTATACACCACACTGTGCATTGAGCAGCACCAGTTAAACAAGGAAAGGGAGCTTATTGAAAGACTAGAGGATCTCAAAGAGCAGCTGGCTCCCCTGGAAAAGGTACGAATTGAGATTAGCAGAAAAGCTGAGAAGAGGACCACTTTGGTGCTATGGGGTGGCCTTGCCTACATGGCCACACAGTTTGGCATTTTGGCCCGGCTTACCTGGTGGGAATATTCTTGGGACATCATGGAGCCAGTAACATACTTCATCACTTATGGAAGTGCCATGGCAATGTATGCATATTTTGTAATGACACGCCAGGA**gttcacctacgaggctctcaaccataggctgttcagcaagaggcaggacaagctgttcaaaagaaacaacttccctaaggaggactacgagaacctcgtgcaggccatcgacaagaaggagaaggagctcaaggagctcgagctcgccacaaagtacgatcacacccaccccggg**GGTGGATCTGGTGGATCTGGTGGATCTATGGATTACAAGGATGACGATGACAAG*

**Combined:**

MAAAAGRSLLLLLSSRGGGGGGAGGCGTLTAGCFPGLGVSRHRQQQHHRTVHQRIASWQNLGAVYCSTVVPSDDVTVVYQNGLPVISVRLPSRRERCQFTLKPISDSVGVFLRQLQEEDRGIDRVAIYSPDGVRVAASTGIDLLLLDDFKLVINDLTYHVRPPKRDLLSHENAATLNDVKTLVQQLYTTLCIEQHQLNKERELIERLEDLKEQLAPLEKVRIEISRKAEKRTTLVLWGGLAYMATQFGILARLTWWEYSWDIMEPVTYFITYGSAMAMYAYFVMTRQE**ftyealnhRlfskrqdKlfkrnnFpkedyenlvqaidkkekelkelelatkydhthpg**GGSGGSGGSMDYKDDDDK*

**Chimera 7:**

ATGGCAGCTGCAGCAGGTAGATCGCTACTGCTACTCCTATCCTCTCGGGGAGGTGGAGGTGGAGGTGCCGGAGGTTGCGGGACGCTGACTGCCGGCTGCTTCCCTGGGCTGGGCGTCAGCCGCCACCGGCAGCAGCAGCACCACCGGACGGTACACCAGAGGATCGCTTCCTGGCAGAATTTGGGAGCTGTTTATTGCAGCACTGTTGTGCCCTCTGATGATGTTACAGTGGTTTATCAAAATGGGTTACCTGTGATATCTGTGAGGCTACCATCCCGGCGTGAACGCTGTCAGTTCACACTCAAGCCTATCTCTGACTCTGTTGGTGTATTTTTACGACAACTGCAAGAAGAGGATCGGGGAATTGACAGAGTTGCTATCTATTCACCAGATGGTGTTCGCGTTGCTGCTTCAACAGGAATAGACCTCCTCCTCCTTGATGACTTTAAGCTGGTCATTAATGACTTAACATACCACGTACGACCACCAAAAAGAGACCTCTTAAGTCATGAAAATGCAGCAACGCTGAATGATGTAAAGACATTGGTCCAGCAACTATACACCACACTGTGCATTGAGCAGCACCAGTTAAACAAGGAAAGGGAGCTTATTGAAAGACTAGAGGATCTCAAAGAGCAGCTGGCTCCCCTGGAAAAGGTACGAATTGAGATTAGCAGAAAAGCTGAGAAGAGGACCACTTTGGTGCTATGGGGTGGCCTTGCCTACATGGCCACACAGTTTGGCATTTTGGCCCGGCTTACCTGGTGGGAATATTCCTGGGACATCATGGAGCCAGTAACATACTTCATCACTTATGGAAGTGCCATGGCAATGTATGCATATTTTGTAATGACACGCCAGGA**gttcacctacgaggctctcaaccataggctgttcagcaagaggcaggacaagctgttcaaaagaaacaacttc**GACCTAGAGAAATACAATCAACTCAAGGATGCAATTGCTCAGGCAGAAATGGACCTTAAGAGACTGAGAGACCCATTACAAGTACATCTGCCTCTCCGACAAATTGGTGAAAAAGATCCCGGGGGTGGATCTGGTGGATCTGGTGGATCTATGGATTACAAGGATGACGATGACAAG*

MAAAAGRSLLLLLSSRGGGGGGAGGCGTLTAGCFPGLGVSRHRQQQHHRTVHQRIASWQNLGAVYCSTVVPSDDVTVVYQNGLPVISVRLPSRRERCQFTLKPISDSVGVFLRQLQEEDRGIDRVAIYSPDGVRVAASTGIDLLLLDDFKLVINDLTYHVRPPKRDLLSHENAATLNDVKTLVQQLYTTLCIEQHQLNKERELIERLEDLKEQLAPLEKVRIEISRKAEKRTTLVLWGGLAYMATQFGILARLTWWEYSWDIMEPVTYFITYGSAMAMYAYFVMTRQE**ftyealnhRlfskrqdKlfkrnn**FDLEKYNQLKDAIAQAEMDLKRLRDPLQVHLPLRQIGEKDPGGGSGGSGGSMDYKDDDDK*

**Chimera 8:**

ATGGCAGCTGCAGCAGGTAGATCGCTACTGCTACTCCTATCCTCTCGGGGAGGTGGAGGTGGAGGTGCCGGAGGTTGCGGGACGCTGACTGCCGGCTGCTTCCCTGGGCTGGGCGTCAGCCGCCACCGGCAGCAGCAGCACCACCGGACGGTACACCAGAGGATCGCTTCCTGGCAGAATTTGGGAGCTGTTTATTGCAGCACTGTTGTGCCCTCTGATGATGTTACAGTGGTTTATCAAAATGGGTTACCTGTGATATCTGTGAGGCTACCATCCCGGCGTGAACGCTGTCAGTTCACACTCAAGCCTATCTCTGACTCTGTTGGTGTATTTTTACGACAACTGCAAGAAGAGGATCGGGGAATTGACAGAGTTGCTATCTATTCACCAGATGGTGTTCGCGTTGCTGCTTCAACAGGAATAGACCTCCTCCTCCTTGATGACTTTAAGCTGGTCATTAATGACTTAACATACCACGTACGACCACCAAAAAGAGACCTCTTAAGTCATGAAAATGCAGCAACGCTGAATGATGTAAAGACATTGGTCCAGCAACTATACACCACACTGTGCATTGAGCAGCACCAGTTAAACAAGGAAAGGGAGCTTATTGAAAGACTAGAGGATCTCAAAGAGCAGCTGGCTCCCCTGGAAAAGGTACGAATTGAGATTAGCAGAAAAGCTGAGAAGAGGACCACTTTGGTGCTATGGGGTGGCCTTGCCTACATGGCCACACAGTTTGGCATTTTGGCCCGGCTTACCTGGTGGGAATATTCCTGGGACATCATGGAGCCAGTAACATACTTCATCACTTATGGAAGTGCCATGGCAATGTATGCATATTTTGTAATGACACGCCAGGAATATGTTTATCCAGAAGCCAGAGACAGACAATACTTACTATTTTTCCATAAAGGAGCCAAAAAGTCACGTTTT**cctaaggaggactacgagaacctcgtgcaggccatcgacaagaaggagaaggagctcaaggagctcgagctcgccacaaagtacgatcacacccaccccggg**GGTGGATCTGGTGGATCTGGTGGATCTATGGATTACAAGGATGACGATGACAAG*

MAAAAGRXLLLLLSSRGGGGGGAGGCGTLTAGCFPGLGVSRHRQQQHHRTVHQRIASWQNLGAVYCSTVVPSDDVTVVYQNGLPVISVRLPSRRERCQFTLKPISDSVGVFLRQLQEEDRGIDRVAIYSPDGVRVAASTGIDLLLLDDFKLVINDLTYHVRPPKRDLLSHENAATLNDVKTLVQQLYTTLCIEQHQLNKERELIERLEDLKEQLAPLEKVRIEISRKAEKRTTLVLWGGLAYMATQFGILARLTWWEYSWDIMEPVTYFITYGSAMAMYAYFVMTRQEYVYPEARDRQYLLFFHKGAKKSRF**pkedyenlvqaidkkekelkelelatkydhthpg**GGSGGSGGSMDYKDDDDK*

**Chimera 9**

ATGGCAGCTGCAGCAGGTAGATCGCTACTGCTACTCCTATCCTCTCGGGGAGGTGGAGGTGGAGGTGCCGGAGGTTGCGGGACGCTGACTGCCGGCTGCTTCCCTGGGCTGGGCGTCAGCCGCCACCGGCAGCAGCAGCACCACCGGACGGTACACCAGAGGATCGCTTCCTGGCAGAATTTGGGAGCTGTTTATTGCAGCACTGTTGTGCCCTCTGATGATGTTACAGTGGTTTATCAAAATGGGTTACCTGTGATATCTGTGAGGCTACCATCCCGGCGTGAACGCTGTCAGTTCACACTCAAGCCTATCTCTGACTCTGTTGGTGTATTTTTACGACAACTGCAAGAAGAGGATCGGGGAATTGACAGAGTTGCTATCTATTCACCAGATGGTGTTCGCGTTGCTGCTTCAACAGGAATAGACCTCCTCCTCCTTGATGACTTTAAGCTGGTCATTAATGACTTAACATACCACGTACGACCACCAAAAAGAGACCTCTTAAGTCATGAAAATGCAGCAACGCTGAATGATGTAAAGACATTGGTCCAGCAACTATACACCACACTGTGCATTGAGCAGCACCAGTTAAACAAGGAAAGGGAGCTTATTGAAAGACTAGAGGATCTCAAAGAGCAGCTGGCTCCCCTGGAAAAGGTACGAATTGAGATTAGCAGAAAAGCTGAGAAGAGGACCACTTTGGTGCTATGGGGTGGCCTTGCCTACATGGCCACACAGTTTGGCATTTTGGCCCGGCTTACCTGGTGGGAATATTCCTGGGACATCATGGAGCCAGTAACATACTTCATCACTTATGGAAGTGCCATGGCAATGTATGCATATTTTGTAATGACACGCCAGGAAT**tcacctacgaggctctcaaccat**AGACAATACTTACTATTTTTCCATAAAGGAGCCAAAAAGTCACGTTTTGACCTAGAGAAATACAATCAACTCAAGGATGCAATTGCTCAGGCAGAAATGGACCTTAAGAGACTGAGAGACCCATTACAAGTACATCTGCCTCTCCGACAAATTGGTGAAAAAGATCCCGGGGGTGGATCTGGTGGATCTGGTGGATCTATGGATTACAAGGATGACGATGACAAG*

MAAAAGRSLLLLLSSRGGGGGGAGGCGTLTAGCFPGLGVSRHRQQQHHRTVHQRIASWQNLGAVYCSTVVPSDDVTVVYQNGLPVISVRLPSRRERCQFTLKPISDSVGVFLRQLQEEDRGIDRVAIYSPDGVRVAASTGIDLLLLDDFKLVINDLTYHVRPPKRDLLSHENAATLNDVKTLVQQLYTTLCIEQHQLNKERELIERLEDLKEQLAPLEKVRIEISRKAEKRTTLVLWGGLAYMATQFGILARLTWWEYSWDIMEPVTYFITYGSAMAMYAYFVMTRQE**ftyealnhR**QYLLFFHKGAKKSRFDLEKYNQLKDAIAQAEMDLKRLRDPLQVHLPLRQIGEKDPGGGSGGSGGSMDYKDDDDK*

**Chimera 10:**

ATGGCAGCTGCAGCAGGTAGATCGCTACTGCTACTCCTATCCTCTCGGGGAGGTGGAGGTGGAGGTGCCGGAGGTTGCGGGACGCTGACTGCCGGCTGCTTCCCTGGGCTGGGCGTCAGCCGCCACCGGCAGCAGCAGCACCACCGGACGGTACACCAGAGGATCGCTTCCTGGCAGAATTTGGGAGCTGTTTATTGCAGCACTGTTGTGCCCTCTGATGATGTTACAGTGGTTTATCAAAATGGGTTACCTGTGATATCTGTGAGGCTACCATCCCGGCGTGAACGCTGTCAGTTCACACTCAAGCCTATCTCTGACTCTGTTGGTGTATTTTTACGACAACTGCAAGAAGAGGATCGGGGAATTGACAGAGTTGCTATCTATTCACCAGATGGTGTTCGCGTTGCTGCTTCAACAGGAATAGACCTCCTCCTCCTTGATGACTTTAAGCTGGTCATTAATGACTTAACATACCACGTACGACCACCAAAAAGAGACCTCTTAAGTCATGAAAATGCAGCAACGCTGAATGATGTAAAGACATTGGTCCAGCAACTATACACCACACTGTGCATTGAGCAGCACCAGTTAAACAAGGAAAGGGAGCTTATTGAAAGACTAGAGGATCTCAAAGAGCAGCTGGCTCCCCTGGAAAAGGTACGAATTGAGATTAGCAGAAAAGCTGAGAAGAGGACCACTTTGGTGCTATGGGGTGGCCTTGCCTACATGGCCACACAGTTTGGCATTTTGGCCCGGCTTACCTGGTGGGAATATTCCTGGGACATCATGGAGCCAGTAACATACTTCATCACTTATGGAAGTGCCATGGCAATGTATGCATATTTTGTAATGACACGCCAGGAATATGTTTATCCAGAAGCCAGAGACAGAC**tgttcagcaagaggcaggac**AAAGGAGCCAAAAAGTCACGTTTTGACCTAGAGAAATACAATCAACTCAAGGATGCAATTGCTCAGGCAGAAATGGACCTTAAGAGACTGAGAGACCCATTACAAGTACATCTGCCTCTCCGACAAATTGGTGAAAAAGATCCCGGGGGTGGATCTGGTGGATCTGGTGGATCTATGGATTACAAGGATGACGATGACAAG*

MAAAAGRSLLLLLSSRGGGGGGAGGCGTLTAGCFPGLGVSRHRQQQHHRTVHQRIASWQNLGAVYCSTVVPSDDVTVVYQNGLPVISVRLPSRRERCQFTLKPISDSVGVFLRQLQEEDRGIDRVAIYSPDGVRVAASTGIDLLLLDDFKLVINDLTYHVRPPKRDLLSHENAATLNDVKTLVQQLYTTLCIEQHQLNKERELIERLEDLKEQLAPLEKVRIEISRKAEKRTTLVLWGGLAYMATQFGILARLTWWEYSWDIMEPVTYFITYGSAMAMYAYFVMTRQEYVYPEARDR**lfskrqd**KGAKKSRFDLEKYNQLKDAIAQAEMDLKRLRDPLQVHLPLRQIGEKDPGGGSGGSGGSMDYKDDDDK*

**HsMCU^CeEDD^**

ATGGCGGCCGCCGCAGGTAGATCGCTCCTGCTGCTCCTCTCCTCTCGGGGCGGCGGCGGCGGGGGCGCCGGCGGCTGCGGGGCGCTGACTGCCGGCTGCTTCCCTGGGCTGGGCGTCAGCCGCCACCGGCAGCAGCAGCACCACCGGACGGTACACCAGAGGATCGCTTCCTGGCAGAATTTGGGAGCTGTTTATTGCAGCACTGTTGTGCCCTCTGATGATGTTACAGTGGTTTATCAAAATGGGTTACCTGTGATATCTGTGAGGCTACCATCCCGGCGTGAACGCTGTCAGTTCACACTCAAGCCTATCTCTGACTCTGTTGGTGTATTTTTACGACAACTGCAAGAAGAGGATCGGGGAATTGACAGAGTTGCTATCTATTCACCAGATGGTGTTCGCGTTGCTGCTTCAACAGGAATAGACCTCCTCCTCCTTGATGACTTTAAGCTGGTCATTAATGACTTAACATACCACGTACGACCACCAAAAAGAGACCTCTTAAGTCATGAAAATGCAGCAACGCTGAATGATGTAAAGACATTGGTCCAGCAACTATACACCACACTGTGCATTGAGCAGCACCAGTTAAACAAGGAAAGGGAGCTTATTGAAAGACTAGAGGATCTCAAAGAGCAGCTGGCTCCCCTGGAAAAGGTACGAATTGAGATTAGCAGAAAAGCTGAGAAGAGGACCACTTTGGTGCTATGGGGTGGCCTTGCCTACATGGCCACACAGTTTGGCATTTTGGCCCGGCTTACCTGGTGGGAATATTCCTGGGACATCATGGAGCCAGTAACATACTTCATCACTTATGGAAGTGCCATGGCAATGTATGCATATTTTGTAATGACACGCCAG**tcatttgaatatccaagtgccagagaaaga**CAATACTTACTATTTTTCCATAAAGGAGCCAAAAAGTCACGTTTTGACCTAGAGAAATACAATCAACTCAAGGATGCAATTGCTCAGGCAGAAATGGACCTTAAGAGACTGAGAGACCCATTACAAGTACATCTGCCTCTCCGACAAATTGGTGAAAAAGATCCCGGGGGTGGATCTGGTGGATCTGGTGGATCTATGGATTACAAGGATGACGATGACAAG

MAAAAGRSLLLLLSSRGGGGGGAGGCGALTAGCFPGLGVSRHRQQQHHRTVHQRIASWQNLGAVYCSTVVPSDDVTVVYQNGLPVISVRLPSRRERCQFTLKPISDSVGVFLRQLQEEDRGIDRVAIYSPDGVRVAASTGIDLLLLDDFKLVINDLTYHVRPPKRDLLSHENAATLNDVKTLVQQLYTTLCIEQHQLNKERELIERLEDLKEQLAPLEKVRIEISRKAEKRTTLVLWGGLAYMATQFGILARLTWWEYSWDIMEPVTYFITYGSAMAMYAYFVMTRQ**sfeypsare**RQYLLFFHKGAKKSRFDLEKYNQLKDAIAQAEMDLKRLRDPLQVHLPLRQIGEKDPGGGSGGSGGSMDYKDDDDK

**HsEMRE^CeNterm^**

**atgaccagcaagaccgtgttccagaacgccttcaagaccttcctggacttcgccatcaacagcctgcccagcacccagggcggcctgaacatcaccgccaccgcccccggcggcgtgggccagaggcccttcaccaacaaggccggc**CTTCTCCGTGTGTTCTCCATTGTGATCCCCTTTCTCTATGTCGGGACACTCATTAGCAAAAATTTCGCGGCACTATTAGAGGAACATGACATTTTTGTTCCAGAGGATGATGATGATGATGAC

**mtsktvfqnafktfldfainslpstqgglnitatapggvgqrpftnkag**LLRVFSIVIPFLYVGTLISKNFAALLEEHDIFVPEDDDDDD

**Cysteine-free MCU**

ATGGCGGCCGCCGCAGGTAGATCGCTCCTGCTGCTCCTCTCCTCTCGGGGAGGTGGAGGTGGAGGTGCAGGAGGTGGAGGTGCGCTGACTGCCGGCGGATTCCCTGGGCTGGGCGTCAGCCGCCACCGGCAGCAGCAGCACCACCGGACGGTACACCAGAGGATCGCTTCCTGGCAGAATTTGGGAGCTGTTTATTACAGCACTGTTGTGCCCTCTGATGATGTTACAGTGGTTTATCAAAATGGGTTACCTGTGATATCTGTGAGGCTACCATCCCGGCGTGAACGCGGTCAGTTCACACTCAAGCCTATCTCTGACTCTGTTGGTGTATTTTTACGACAACTGCAAGAAGAGGATCGGGGAATTGACAGAGTTGCTATCTATTCACCAGATGGTGTTCGCGTTGCTGCTTCAACAGGAATAGACCTCCTCCTCCTTGATGACTTTAAGCTGGTCATTAATGACTTAACATACCACGTACGACCACCAAAAAGAGACCTCTTAAGTCATGAAAATGCAGCAACGCTGAATGATGTAAAGACATTGGTCCAGCAACTATACACCACACTGCGCATTGAGCAGCACCAGTTAAACAAGGAAAGGGAGCTTATTGAAAGACTAGAGGATCTCAAAGAGCAGCTGGCTCCCCTGGAAAAGGTACGAATTGAGATTAGCAGAAAAGCTGAGAAGAGGACCACACTAGTGCTATGGGGTGGCCTTGCCTACATGGCCACACAGTTTGGCATTTTGGCCCGGCTTACCTGGTGGGAATATTCCTGGGACATCATGGAGCCAGTAACATACTTCATCACTTATGGAAGTGCCATGGCAATGTATGCATATTTTGTAATGACACGCCAGGAATATGTTTATCCAGAAGCCAGAGACAGACAATACTTACTATTTTTCCATAAAGGAGCCAAAAAGTCACGTTTTGACCTAGAGAAATACAATCAACTCAAGGATGCAATTGCTCAGGCAGAAATGGACCTTAAGAGACTGAGAGACCCATTACAAGTACATCTGCCTCTCCGACAAATTGGTGAAAAAGAT

MAAAAGRSLLLLLSSRGGGGGGAGGGGALTAGGFPGLGVSRHRQQQHHRTVHQRIASWQNLGAVYYSTVVPSDDVTVVYQNGLPVISVRLPSRRERGQFTLKPISDSVGVFLRQLQEEDRGIDRVAIYSPDGVRVAASTGIDLLLLDDFKLVINDLTYHVRPPKRDLLSHENAATLNDVKTLVQQLYTTLRIEQHQLNKERELIERLEDLKEQLAPLEKVRIEISRKAEKRTTLVLWGGLAYMATQFGILARLTWWEYSWDIMEPVTYFITYGSAMAMYAYFVMTRQEYVYPEARDRQYLLFFHKGAKKSRFDLEKYNQLKDAIAQAEMDLKRLRDPLQVHLPLRQIGEKDPGGGSG
